# Human iPSC banking: barriers and opportunities

**DOI:** 10.1186/s12929-019-0578-x

**Published:** 2019-10-28

**Authors:** Ching-Ying Huang, Chun-Lin Liu, Chien-Yu Ting, Yueh-Ting Chiu, Yu-Che Cheng, Martin W. Nicholson, Patrick C. H. Hsieh

**Affiliations:** 1 Institute of Biomedical Sciences, Academia Sinica, Taipei, Taiwan; 2 Graduate Institute of Medical Genomics and Proteomics and Graduate Institute of Clinical Medicine, National Taiwan University College of Medicine, Taipei, Taiwan; 3 Cardiovascular Surgery Division, Department of Surgery, National Taiwan University Hospital, Taipei, Taiwan

**Keywords:** Induced pluripotent stem cell (iPSC), Cell bank, Personalized medicine

## Abstract

The introduction of induced pluripotent stem cells (iPSCs) has opened up the potential for personalized cell therapies and ushered in new opportunities for regenerative medicine, disease modeling, iPSC-based drug discovery and toxicity assessment. Over the past 10 years, several initiatives have been established that aim to collect and generate a large amount of human iPSCs for scientific research purposes. In this review, we compare the construction and operation strategy of some iPSC banks as well as their ongoing development. We also introduce the technical challenges and offer future perspectives pertaining to the establishment and management of iPSC banks.

## Introduction

Since the generation of induced pluripotent stem cells (iPSCs) by Shinya Yamanaka and his colleagues in 2006 [1, 2], there has been an ever-growing interest in exploiting the full potential of these extraordinary cells. In culture, iPSCs are able to self-renew and differentiate into any cell type from all three germ layers (ectoderm, mesoderm, and endoderm), and importantly, use of iPSCs avoids the ethical issues associated with embryonic stem cells. Furthermore, the development of iPSC technology allows for an almost unlimited amount of either healthy or disease-specific human pluripotent stem cells. Obtaining such cells is a major hurdle when employing primary, patient-derived disease-affected cell types, which represent the ‘gold standard’ for disease modeling [3]. Due to these characteristics, iPSCs hold great promise for use in biomedical research and development.

Unfortunately, however, the high cost of generating and validating iPSCs hinders their use by many researchers. Therefore, there is a need for cell banks which provide high-quality iPSCs to researchers who would otherwise be unable to generate and characterize these cells in their own labs. This review provides a comprehensive comparison of the current iPSC banks worldwide. First, we briefly review the applications of iPSCs and summarize their generation, characterization and quality control. Then, we provide a comprehensive review of the state of the major existing iPSC banks worldwide and the current barriers being faced in the field of iPSC banking.

### Applications of iPSCs

The self-renewal property of iPSCs in culture allows for extensive studies employing donor-derived, healthy and diseased cell lines. Multiple diseased iPSC lines have been generated allowing the study of human disease phenotypes which are currently difficult to obtain in animal models, making iPSCs an attractive option for use in drug screening and toxicity studies, drug development, human disease modeling, personalized medicine, and cell-based therapy.

It is estimated that 27, 14 and 7% of drugs fail in clinical trials due to adverse effects on the heart, liver and central/peripheral nervous systems, respectively [4]. This is, in part, due to the use of animal models for drug screening which poorly replicate the human system [5]. Using human iPSCs for drug screening avoids cross-species differences before they are taken to clinical trials. This not only greatly reduces the number of animals used in drug screening studies but also improves the success rates in clinical trials. Thus, iPSCs from both healthy and diseased patients are gaining traction as the preferred cell of choice for drug screening and toxicity studies. Recently, it was shown that amyotrophic lateral sclerosis patient iPSC-derived motor neurons displayed hyperexcitability and reduced survival in culture. The researchers showed that this could be corrected by a potassium channel agonist already approved by the FDA allowing the drug to go directly into phase II clinical trials for the treatment of amyotrophic lateral sclerosis without the need for animal studies [6]. Many other drug screening studies can be found for diseases such as Parkinson’s disease [7], retinitis pigmentosa [8], and pulmonary arterial hypertension [9], to name a few. Further information can be found in Leitt et al. 2018 which reviewed the current drug screening studies for human diseases using iPSCs [3].

In recent years, researchers have taken iPSCs from the lab to the clinic. The use of iPSCs in regenerative medicine provides an exciting opportunity for the clinical translation of this technology, whereby patient-specific iPSCs are generated for autologous transplantation to repair or replace injured tissues. To facilitate iPSC-based research and clinical therapies in Japan, CiRA was selected as the main center to conduct “iPSC stock development projects for regenerative medicine”. Keio University, CiRA, RIKEN, and Osaka University play roles as clinical application research centers, which aim to promote iPSC-based cell therapy [10]. In 2014, RIKEN carried out the first clinical trial of iPSC transplantation by transplanting iPSC-derived retinal pigment epithelial cells to treat macular degeneration [11]. As a result, further macular degeneration was not observed and the patient reported improved vision [11]. Moreover, Professor Takahashi and colleagues from Kyoto University/CiRA successfully implanted iPSC-derived dopaminergic neurons into the brain of a Parkinson’s patient. This was the first clinical trial employing iPSCs to treat Parkinson’s disease. Takahaski reported that the patient is recovering well, and that they plan to treat a further 6 patients if no complications arise [12]. In addition, Dr. Sawa and his team from Osaka University received approval to implant iPSC-derived cardiac cell sheet onto three heart failure patients [13]. More recently, the Japanese government’s health ministry has approved Dr. Okano and colleagues from Keio University School of Medicine to inject iPSC-derived neural cells into four patients with spinal cord injuries [14]. Although these studies are still in their infancy, regenerative medicine and cell replacement therapy employing iPSCs may soon be more widely available.

### Generation and characterization of iPSCs

#### Cell sources

In 2006, Yamanaka and colleagues showed that mouse fibroblasts can be reprogrammed into iPSCs when retrovirally transduced with defined factors [1]. The following year, human fibroblasts were successfully reprogrammed into iPSCs using the same [2] or similar factors [15]. From this point on, fibroblasts were the most extensively used cell-type for iPSC generation due to their ease of handling and ready availability from skin biopsy. Theoretically, all actively dividing somatic cells are capable of being reprogrammed into iPSCs, such as peripheral blood mononuclear cells, fibroblasts, T cells, B cells and hepatocytes [2, 16–20] (Table 1). Moreover, even the less proliferative cardiomyocytes can be reprogrammed into iPSCs [21, 22] suggesting that most cell types can be reprogrammed into iPSCs. Among these cells, PBMCs are more advantageous over fibroblasts since blood extraction is minimally invasive and requires a small volume of 2–6 mL. Moreover, PBMCs can be reprogrammed immediately after sample collection [23]. However, fibroblasts are obtained from a patients’ skin punch biopsy which is, in contrast, a more invasive procedure. Isolated cells must then be cultured, expanded and passaged before reprogramming. Therefore, PBMCs have become the most common cell source for iPSCs generation.

**Table 1:** Brief overview of iPSC generation and characterization

Bank Name	Cell Sources	Reprogramming Methods	Characterization methods
California Institute for Regenerative Medicine (CIRM)	Blood cells (1148) Fibroblasts (263)	Episomal vectors	Characterization is carried out by Coriell and FCDI
Coriell Institute for Medical Research (Coriell)	Fibroblasts Blood cells	Retrovirus (40%) Sendai virus (30%) Episomal vectors (27%) Lentivirus (3%)	*General*: post-thaw viability, mycoplasma detection, identity match, karyotyping, sterility, IF analysis of pluripotency *Specific*: Sendai virus clearance, Alkaline Phosphatase analysis of pluripotency, 3-germ-layer EB/teratoma differentiation, loss of episomal plasmids
Fujifilm Cellular Dynamics International (FCDI)	Blood cells (1148) Fibroblasts (263)	Episomal vectors	*General*: mycoplasma detection, identity match, karyotyping, sterility, pluripotency analysis, loss of episomal plasmids
Center for iPS Cell Research and Application (CiRA)	PBMC Cord blood Dental pulp	Episomal vectors Retrovirus	CiRA characterizes clinical-grade iPSCs by: post-thaw viability, mycoplasma detection, identity match, karyotyping, sterility, Sendai virus clearance, flow/microarray analysis of pluripotency, virus screening, SNV/INDEL/CNV, endotoxin
European Bank for induced pluripotent Stem Cells (EBiSC)	Fibroblasts (> 75%)	Sendai virus (> 80%) Episomal vectors Retrovirus Transposon Lentivirus mRNA	*General*: post-thaw viability, mycoplasma detection, identity match, karyotyping, Sendai virus clearance *Specific*: IF/flow cytometry /Pluri analysis of pluripotency, 3-germ-layer EB differentiation, virus screening, CNV, RNA-seq, exome seq, genotyping array, methylation array
Human Induced Pluripotent Stem Cell Initiative (HipSci)	PBMC (30) Fibroblasts (805)	Sendai virus	*General*: post-thaw viability, mycoplasma detection, identity match, Sendai virus clearance, Pluri analysis of pluripotency, CNV *Specific*: RNA-seq, exome-seq, genotyping array, methylation array, expression array, whole genome-seq, mass spectrometry, cellular phenotyping
Human Disease iPSC Consortium Resource Center (Taiwan Human Disease iPSC Consortium)	PBMC Fibroblasts	Sendai virus	*General*: post-thaw viability, mycoplasma, identity match, karyotyping, Sendai virus clearance, IF/flow cytometry /RT-PCR analysis of pluripotency, 3-germ-layer EB/teratoma differentiation, CNV, SNP genotyping
Institute of Physical and Chemical Research (RIKEN)	PBMC Cord blood Skin	Sendai virus (40%) Retrovirus (30%) Episomal vectors (30%)	*General*: post-thaw viability, mycoplasma detection, identity match *Specific*: karyotyping, ability to differentiate into specific cell type
Korean National Stem Cell Bank (KSCB)	Fibroblasts	Sendai virus mRNA	*General*: mycoplasma, identity match, karyotyping, Sendai virus clearance, IF/RT-PCR analysis of pluripotency, 3-germ-layer EB/teratoma differentiation
WiCell Research Institute (WiCell)	Blood cells	Sendai virus (> 50%) Episomal vectors (> 25%) Lentivirus Retrovirus	*General*: post-thaw viability, mycoplasma detection, identity match, karyotyping, sterility, *Specific*: spectral karyotyping, FISH, SNP microarray

Note 1: Reprogramming methods are compiled from currently available cell lines. Cells not open on shelf or at the status of generation are not included in the percentage counts

Note 2: Owing that iPSC banks collected cells from numerous organizations/institutions, characterization items may differ from line to line whilst deposited at the same bank. To acquire more comprehensive characterization items, it is suggested to look directly online at cell catalog of the bank. Characterization items summarized here list out “*General*” inspections that each cell line must undergo, and “*Specific*” examinations” that only performed on selected or applied lines

Immunofluorescence(IF)/ Single nucleotide variation (SNV)/Insertion and/or deletion (INDEL)/ Reverse transcription-PCR (RT-PCR)/Fluorescence in situ hybridization (FISH)/ Single nucleotide polymorphism (SNP)/ Embryoid body (EB)

#### Reprogramming methods

At first, retrovirus and lentivirus were extensively used to generate iPSCs. However, these two viruses can randomly integrate into the host genome and increase the risk of mutagenesis. To avoid genome integration, new methods were developed and optimized such as adenovirus [24], Sendai virus [19, 25, 26], plasmid vectors [27–29], piggyBac transposons [30–32], synthesized RNAs [33], and use of recombinant proteins [34] (Table 1). Among these, Sendai virus is the most widely applied reprogramming method due to two characteristic advantages. First, Sendai virus is an RNA virus that does not enter the nucleus, which means not integrating into the host genome [25]. Second, the cells can be reprogrammed at an efficiency of 0.1% for fibroblasts and 0.1% for PBMCs [26]. Therefore, many laboratories and biobanks use Sendai virus to reprogram a wide range of somatic cells [35–37] due to its high efficacy and convenience.

#### Factor selection

In addition to the Yamanaka factors (Oct3/4, Sox2, Klf4, and c-Myc), Thomson’s factors (Oct3/4, Sox2, Nanog, and Lin28) can also act as master regulators to reprogram somatic cells into iPSCs. Oct3/4 is the transcription factor that controls the maintenance and regaining of stem cell pluripotency [38]. Sox2 governs pluripotency through the regulation of Oct3/4 expression [39] while Nanog orchestrates the transcriptional network with Oct3/4 and Sox2. Klf4 exerts an anti-apoptotic effect leading to self-renewal of iPSCs [40] and activates Sox2 [41]. Lin28, a highly conserved RNA-binding protein, regulates mRNA translation and also controls self-renewal of stem cells [42]. c-Myc facilitates histone acetylation, resulting in an open chromatin structure, allowing Oct3/4 and Sox2 to access their genome loci [34, 43]. However, c-Myc has been reported to act as a proto-oncogene causing various cancers. Therefore, L-Myc, another Myc family member with less tumorigenicity, may be a substitution for c-Myc [44].

#### Characterization of iPSCs

According to the suggestions laid out by the International Stem Cell Banking Initiative, there are specific criteria that should be met before banking an iPSC line [45]. Most bio-banks have common characterization methods for establishing iPSC lines which include: (1) embryonic-like morphology observation; (2) transgene silencing after reprogramming; (3) pluripotency assessment including alkaline phosphatase assay or detection of pluripotent and renewal markers such as TRA-1-60, TRA-1-81, Nanog, Oct4; (4) differentiation potential both in vitro (embryoid body formation) and in vivo (teratoma formation); (5) karyotype analysis to indicate chromosomal abnormalities; (6) identity confirmation by DNA fingerprinting and short tandem repeat-PCR; and (7) microbiological assay to ensure the culture is free of any possible biological contaminants (Table 1). It is important for cell banks to provide useful characterization data and information for either research-grade or clinical-grade iPSCs.

### Quality assurance and quality control of iPSC banks

**Fig. 1:**
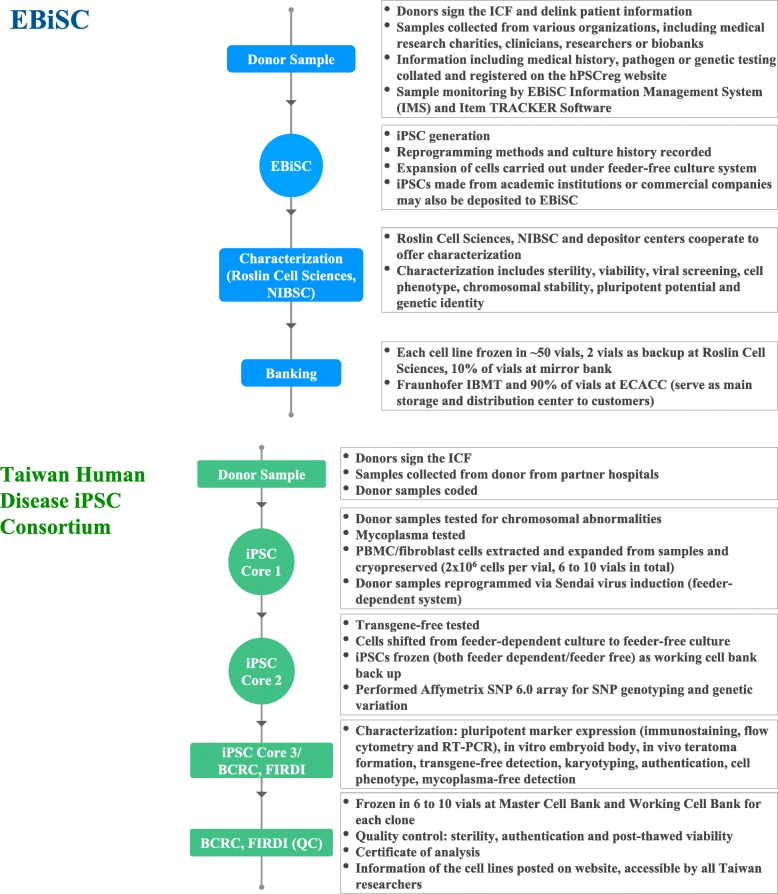
Workflow of EBiSC and Taiwan Human Disease iPSC Consortium

To generate, deposit and deliver high-quality iPSCs seamlessly to institutes and customers requires extensive experience, effort, and stringent management. In a stem cell bank, a well established and standardized quality assurance (QA) process is required to ensure banked iPSC pluripotency and quality; quality control (QC) is also important to ensure the quality of banked iPSC vials. Herein, we briefly introduce established SOPs at two iPSC banks, the European Bank for induced pluripotent Stem Cells (EBiSC) and the Human Disease iPSC Consortium in Taiwan (Fig. 1).

#### European Bank for induced pluripotent stem cells (EBiSC)

EBiSC launched its Hot Start project in 2014 in collaboration with several public and private organizations across Europe. Babraham Research Campus located in Cambridge, UK, is the main facility responsible for cell expansion, QC, and characterization. The European Collection of Authenticated Cell Cultures (ECACC) of Public Health England, also in the UK, is the major bank for cell storage and distribution to worldwide users while Fraunhofer-Institut für Biomedizinische Technik (IBMT) in Saarbrücken, Germany, is a mirror storage bank of ECACC [46].

With years of experience, EBiSC is renowned for its rigorous standardized pipelines and serves as a good foundation for initiatives of future iPSC banks [47]. Upon receiving donor samples, with donor consent attached, pathogen/genetic testing is performed. Once passed, the workflow continues onto iPSC generation, deposit, and distribution. To insure all central or ancillary facilities carry out the same procedures while handling the cells, standard protocols have been established both in text and video formats [48, 49]. Routine training courses are also held to ensure inter-institutional consistency.

Once iPSC generation is completed, a series of characterization assays are undertaken to investigate sterility from mycoplasma and bacteria, cell phenotype using flow analysis and/or naked eye observation, chromosomal stability (karyotype by G-banding), genetic identity (STR analysis), and pluripotent potential (three germ layer differentiation). Of particular note, EBiSC plans to introduce new characterization technology, such as automatic imaging to replace naked eye observation of aneuploidies, and use of KaryoLite BoBs instead of traditional G-banding as it is easy to interpret KaryoLite BoBs results and it is a rapid method to detect aneuploidies. They also plan to employ TaqMan array plates to assess pluripotency [50] all with the aim of improving characterization efficiency.

Banking cells with standardized procedures can guarantee more consistent high-quality and post-thaw survival rate of iPSCs. EBiSC graphed out a detailed process of cell banking [50], similar to the characterization methods mentioned above. Additional banking processes include culturing cells in antibiotic-free medium for 3 passages and subsequent assays to verify that the cells are free from any reprogramming vectors.

On average, 50 vials are generated per cell line. Approximately 90% of the vials are deposited at the ECACC and 10% are stored at Roslin Cell Sciences and IBMT as a backup. To track current distribution status, Item TRACKER Software is implemented to locate individual vials and enhance the vials traceability. To improve inter-institutional communication and management, Information Management System (IMS) was developed by EBiSC to log cell line information and status. Users may also use the IMS online catalog to request a data package of each cell line and order via an E-commerce tool to obtain cells from ECACC. Elegantly designed, IMS also serves as an integration platform of user-generated data from various sources.

Transferring iPSC vials across institutes requires clear annotation and a thoroughly-labelled system. EBiSC has created its own rules for labelling and identifying cells, providing information such as origin of depositor, iPSC line, donor, clone and subclone number. Labels also include batch/catalog numbers and a 2D QR-Code. Each code is assigned to a specific cell ID and is compatible with existing automated cryostorage devices [47, 50].

Automation of the pipeline is a future goal for all iPSC banks. Artificial intelligence-combined machine arms can precisely monitor cell morphology and confluency in a timely manner, and operate cells by exactly the same built-in programs. This can greatly increase reproducibility among batches of experiments and decrease labor-intensive activities. EBiSC leads the automation infrastructure by establishing an automated cryopreservation system at the cell bank in IBMT [50]. Other systems are under development, including those at the Babraham Research Campus, which aim to automate cell culturing and expansion.

#### Taiwan Human Disease iPSC Consortium

Founded in 2015 by the Taiwan Ministry of Science and Technology, five laboratories were brought together to form the Taiwan Human Disease iPSC Consortium including four iPSC cores located in the Institute of Biomedical Science (IBMS) of Academia Sinica, National Taiwan University Hospital, Taipei Veteran General Hospital, and National Health Research Institutes (2015–2017). These cores are the main facilities responsible for iPSC generation and differentiation into different cell types such as cardiomyocytes and retinal pigment epithelial cells, while the Food Industry Research and Development Institute (FIRDI) is responsible for cell expansion, QC, characterization, and cell banking. IBMS has been the leader and main administrative organization of the consortium since 2015. In June 2019, FIRDI has transferred the duty of iPSC characterization to IBMS, as such, FIRDI is now only responsible for cell banking.

Samples are extracted from donors after an informed consent form is signed; they are then cryopreserved in the collaborating hospitals. All donor samples are coded using a delinked number; however, other donor information such as age, gender, and specific genetic mutations are provided. Apart from this information, all other personal information is excluded. Upon receipt, donor samples are tested to confirm that they are free of mycoplasma, at which point, iPSCs are generated using Sendai virus at the iPSC cores. In addition, another 10 mL of blood sample is sent to a centralized characterization core at FIRDI where a chromosomal integrity test is performed. Each donor’s sample has approximately 6 to 10 extra vials cryopreserved in liquid nitrogen with each containing 2 × 10^6^ cells as a backup at the iPSC core facility. To confirm that standardized operation protocols are consistently followed within different iPSC cores, routine training courses are held within the core facility and inter-core facilities, and all frontline workers from each iPSC core have a laboratory meeting every other month.

Once generated, the iPSCs are maintained for 8 passages at which point RNA is collected and tested for the presence of Sendai virus using RT-PCR. For every iPSC line, three Sendai virus-free clones are selected, shifted from a feeder-dependent culturing system (inactivated mouse embryonic fibroblast) to a feeder-free culturing system. Approximately 10 vials of each iPSC clone are frozen and stored in the working cell bank of the iPSC Core. The virus-free iPSCs are then shipped to FIRDI for iPSC characterization, where iPSC lines are tested for their freeze-thaw viability.

Each clone is expanded and cryopreserved in the Master Cell Bank at the Bioresource Collection and Research Center (BCRC) using standardized procedures. One vial of the iPSCs is defrosted, expanded, then further cryopreserved into a working cell bank of 10 vials. Subsequently, a series of characterization assays is performed on the iPSCs defrosted from the working cell bank. iPSC characterization assays are performed for QA, which includes tests of pluripotent potential (embryoid body formation and teratoma formation) and iPSC identification (RT-PCR, immunofluorescence, and flow cytometry). Quality control assays include sterility testing (testing for the presence of mycoplasma, bacterium, and fungi), genetic identity (STR-PCR analysis), and chromosomal integrity (karyotyping by G-banding). In addition, whole genome single nucleotide polymorphism (SNP) array is performed (Affymetrix Genome-Wide SNP Array 6.0) to identify genetic variation, caused by the reprogramming process, in these iPSCs (such as copy number variation (CNV), SNP or loss of heterozygosity). Upon completion of QA/QC assays, a certificate of analysis is generated tailored for each cell line. To ensure ease of distribution across institutes, a barcode annotation system is used to label all cell lines. Information pertaining to the iPSCs generated, along with the complete certificate of analysis, is available on the BCRC’s website available to researchers in Taiwan.

### Existing iPSC banks and resource sharing

Most institutes offering iPSC generation, characterization and banking are non-profit organizations and are mainly government funded. With the scale and influence of the major iPSC banks, it seems that only governments have the ability to orchestrate the collaboration between numerous patient donors and characterization facilities. These institutes aim to better the development of stem cell research and provide specific disease cell lines for academic and industrial research (Table 2).

**Table 2:** Brief overview of iPSC banks worldwide

Bank Name	Location	Profit Type	Ownership	Types of Diseases	Number of iPSC Lines	Investment (USD)	Related Publication
California Institute for Regenerative Medicine (CIRM)	USA	Non-profit	Government-owned	23	1556	$3000,000	[51–53]
Coriell Institute for Medical Research (Coriell)^a^	USA	Non-profit	Government-owned	40	91	$4,250,000 from NIH $10,000,000 from CIRM	[54–56]
Fujifilm Cellular Dynamics International (FCDI)^b^	USA	Profit	Owned by Fujifilm	N/A	N/A	$16,000,000 and shared grant of $6,300,000 from CIRM	[57–61]
Center for iPS Cell Research and Application (CiRA)	Japan	Non-profit	Government-owned	10	22	$27,383,000	[62–67]
European Bank for induced pluripotent Stem Cells (EBiSC)	Europe	Non-profit	European Commission, private enterprises	36	815	EBiSC project- $38,423,189 EBiSC2 project- $9,931,047	[68–71]
Human Induced Pluripotent Stem Cell Initiative (HipSci)	UK	Non-profit	Medical Research Council, Wellcome Trust	15	835	$20,500,000	[72–77]
Human Disease iPSC Consortium Resource Center (Taiwan Human Disease iPSC Consortium)	Taiwan	Non-profit	Government-owned	20	78	N/A	[78–94]
Institute of Physical and Chemical Research (RIKEN)	Japan	Non-profit	Government-owned	68	480	$24,862,180 for all departments	[95–98]
Korean National Stem Cell Bank (KSCB)	Korea	Non-profit	Government-owned	0	15	N/A	[99, 100]
WiCell Research Institute (WiCell)	USA	Non-profit	University of Wisconsin-Madison	58	1316	N/A	[101–104]

^a^Coriell has its own iPSC depositories: (1) NIGMS Human Genetic Cell Repository (15 healthy donor-derived iPSC lines and 37 diseased iPSC lines); (2) NIA Aging Cell Repository (3 diseased iPSC lines); and (3) Allen Institute for Cell Science. By using CRISPR technology, the Allen Institute generates 36 fluorescent-tagged (EGFP/RFP) iPSC lines from one healthy donor, which produces a potent research tool by tagging different cellular organelles, proteins and compartments. Owing to Coriell’s expertise in cryopreservation and banking, Coriell was awarded with $10 million grant by CIRM to redeposit donor samples and iPSC lines from the Human iPSC Initiative project

^b^Founded by James Thomson and now acquired by Fujifilm, FCDI is renowned for its episomal reprogramming technique. Unlike other banks, FCDI generates diverse iPSC-derived differentiated/progenitor cells that are available for purchase: iCells and donor-specific MyCells. Although FCDI does not function as an iPSC bank that offers iPSC lines, it partners with CIRM in carrying out two iPSC projects: (1) CIRM gave FCDI a grant of $16 million to generate 3 iPSC lines per person from 3000 healthy and diseased donors from the Human iPSC Initiative project; (2) the FCDI and Medical College of Wisconsin (MCW) together received $6.3 million from CIRM. FCDI aims to generating 250 iPSC lines and their cardiomyocytes derived from Caucasian and African-American donors to study left ventricular hypertrophy

#### California Institute for Regenerative Medicine (CIRM)

CIRM was founded in 2004 by the California state government with the intent to establish a state-of-the-art organization for regenerative research operating with US $3 billion in state government funding [105]. It not only participates in the reprogramming of iPSCs from donor blood, but also has a rigorous in-house iPSC characterization and QC workflow. It uses SNP microarray to identify variance from the donor genome in order to score for chromosomal integrity. The generated iPSCs then go through mRNA expression analysis, which has replaced the traditional teratoma assays, to identify the expression of stemness markers. The iPSC lines are then compared to the donor through genotyping requiring less than two mismatches in the 48 SNPs to pass QC. To ensure the removal of reprogramming transgenes, PCR is performed to detect the residual plasmids at passage 5. Finally, the cell lines are tested for mycoplasma in-house and overall sterility using a third-party service [106]. As of now, CIRM is responsible for multiple funding awards from research to clinical trials. A stem cell bank was established as part of the institution, and since then, CIRM has generated 1556 individual iPSC lines with 23 unique disease types including, but not limited to, heart diseases such as cardiomyopathies, and neurodegenerative disease such as Alzheimer’s disease. The majority of the cell lines were generated from donor’s B lymphocytes with around 17% using fibroblasts as the cell source [107]. CIRM collaborates with Fujifilm Cell Dynamics and the Coriell Institute in cell derivation and banking. In 2017, CIRM invested US $32 million in obtaining donor samples, cell line generation, characterization, cell banking, and overall maintenance [108] .

#### Center for iPS cell research and application (iCeMS), Kyoto University

In 2008, Kyoto University established a new research institute, iCeMS. In March 2010, shortly after initiating iCeMS, Kyoto University announced the foundation of the Center for iPS Cell Research and Application (CiRA) in collaboration with the Kyoto Prefectural Government and RIKEN BioResource Research Center (BRC). Lead by Dr. Shinya Yamanaka, CiRA aims to further explore the potential of iPSCs as a new resource for drug discovery and regenerative medicine [109]. Each year, CiRA receives, on average, US $27.383 million from the donations of individuals, corporations, and organizations, and in 2015 they had a balance of US $83.9 million in their iPSC research fund [110]. As a world-leading research institute of iPSC technology, CiRA has founded the Facility for iPS Cell Therapy, which is responsible for generating clinical-grade iPSCs and has deposited 22 human iPSC lines, including 12 normal iPSC lines and 10 diseased iPSC lines comprised of three unique diseases.

#### EBiSC

The EBiSC was initially launched by the Hot Start project [47] and received US $38.4 million in funding. It is comprised of numerous sectors including consulting enterprises, iPSC generation and characterization, storage and distribution, legal and ethics, and bio-engineering and automation groups that are spread across European nations. High-standard SOPs for iPSC QA and QC are being established and shared by EBiSC [47]. Currently, the EBiSC offers 306 normal and 482 diseased iPSC lines, including 27 CRISPR-mediated isogenic controls now available to researchers worldwide [111]. In March 2019, EBiSC embarked on a second project “EBiSC2”, with US $9.93 million in funding, aiming to provide: (1) a more complete catalog of CRISPR-mediated isogenic controls or gene-modified lines; (2) hiPSC-derived progenitor cells; and (3) ready-to-use screening platforms between control and diseased lines. In order to generate a large quantity while maintaining constant cell quality, automation of the pipeline is now underway. Users not only have access to iPSC lines but also stringent online filmed/documented protocols set up by the EBiSC.

#### Korean Society for Cell Biology (KSCB)

The KSCB is an organization for iPSC and ESC line banking and distribution that operates under the Korea National Institute of Health. Researchers can apply to access the 15 listed iPSC lines, most of which are from healthy donors’ fibroblasts; however, there are a number of cell lines being developed using RNA-based gene delivery to generate cytogenetic abnormalities. KSCB and its stem cell bank are completely government-owned and funded [112].

#### Human induced pluripotent stem cell initiative (HipSci)

Located in the UK and funded by the Medical Research Council/Wellcome, with a total of US $20.5 million in funding, HipSci has collected 835 donor samples, the majority of which have a British background, including 15 disease lines [113, 114]. The organization heavily utilizes the Cytotune 2.0 Sendai Virus Kit to generate iPSC lines and collaborates with ECACC/EBiSC to deposit/distribute cells. The advantage of HipSci over other biobanks is their extensive effort in characterizing iPSC lines. Genetic and genomic assays (RNA seq/DNA methylation/whole genome seq/exome seq), proteomic assays, and cellular phenotyping assays are included in the pipeline. HipSci has 496 healthy donor-derived iPSC lines that can be used for identifying genetic variations that occur in the general population. Researchers can access these data online and apply for use; however, they currently do not offer customized iPSC generation [115].

#### RIKEN – BioResource research center (BRC)

To date, RIKEN BRC holds an iPSC bank with approximately 480 normal iPSC lines and 68 unique diseased iPSC lines [116]. In addition to iPSC banking, RIKEN BRC is focusing on the development of iPSC characterization and iPSC-based drug discovery. With its collaboration with Kyoto University, they formed the CiRA in 2008, which focuses on the iPS Cell Stock for Regenerative Medicine and aims to provide clinical grade iPSCs to industry and research institutes [117].

#### Taiwan Human Disease iPSC Consortium

Taiwan Human Disease iPSC Consortium is the first, and the only iPSC resource center in Taiwan that aims to provide iPSC generation, characterization, and an iPSC banking service. The consortium was founded in 2015 under the Taiwan government’s National Research Program for Biopharmaceuticals project. In 2017, the consortium was transferred into another program called the National Core Facility for Biopharmaceuticals. For the past three years, the consortium has received funding from the National Research Program for Biopharmaceuticals and the National Core Facility for Biopharmaceuticals program which totals US $2.1 million. Blood or fibroblast samples are collected and sent to the iPSC cores to be generated into iPSC lines, which are subsequently sent to FIRDI for QC and iPSC banking [118]. To date, 78 Sendai virus reprogrammed iPSC lines have been generated by the Taiwan iPSC Consortium consisting of 11 normal and 67 diseased iPSC lines. As of February 2019, there have been 20 individual disease types banked in the Taiwan iPSC Consortium. Furthermore, out of the 78 iPSC lines, 57 are feeder-free iPSC lines and 21 are feeder-dependent iPSC lines, all of which are accessible to all researchers in Taiwan via the BCRC website.

#### WiCell

As a supporting organization of the University of Wisconsin-Madison, WiCell, established in 1999, is a non-profit organization focusing on the betterment of stem cell research. Starting with banking and distributing embryonic stem cell (ESC) lines, WiCell quickly expanded their collection into iPSC lines [119]. WiCell has generated and characterized 1316 iPSC lines from donor blood with 58 individual disease types across the spectrum from sickle cell anemia to mental illness. These cell lines are readily available to both academic and industrial groups. WiCell offers services including cell line generation, mycoplasma detection, karyotyping, cell banking, and other services. Other than stem cell generation, WiCell also offers services in cell line banking, operating under good manufacturing practice conditions with modified iPSC lines and differentiated cell lines readily available for purchase [119].

### Barriers in iPSC application

Over the last decade, numerous studies of patient-specific iPSC-based disease modeling have been reported; however, the majority of these studies based their conclusions on employing one to a few patient-derived iPSC lines, their isogenic controls, and normal iPSC lines. Various mutations can occur in the same gene and lead to different phenotypes in different individuals. Also, genetic background, epigenetic modifications, and variation amongst clones in iPSC lines can affect results observed by researchers. Thus, a large cohort of diseased iPSCs is needed to understand the underlying mechanism for each disease. To this end, projects for large-scale collection of iPSCs from normal and diseased individuals have been growing over the past ten years. The value of iPSC biobanks and resources are related to the information and QC that are provided to the users. This section aims to describe the hurdles faced in translating iPSC applications into the clinic. Although a few clinical studies based on iPSC derivatives are ongoing, QC, reproducibility, and immunogenicity are the biggest barriers for iPSC utility.

#### Immunogenicity

The discovery of iPSC-based technology offers a promising cell source for autologous cell transplantation for various degenerative diseases without side effects from immunosuppression and allograft rejection. In 2011, Zhao and colleagues reported that injection of iPSC-derived teratoma into syngeneic host mice resulted in immune rejection. This study raises a concern regarding the use of autologous iPSC transplantation for cell therapy and the immunogenicity of undifferentiated iPSCs [120]. Almeida et al. tried to compare the immunogenicity of undifferentiated autologous iPSCs, iPSC derivatives, and syngeneic somatic cells after cell transplantation; they demonstrated that autologous iPSC derivatives could engraft into tissue without using immune suppression and elicited a tolerogenic immune response very similar to the syngeneic somatic cell group. However, the autologous undifferentiated iPSC graft was rejected by the recipient with lymphocytic infiltration [121]. This work has proved that iPSC derivatives result in loss of immunogenicity. Moreover, Embrog et al. transplanted autologous iPSC-derived neural progenitor cells into the non-human primate brain and six months after transplantation, found no infiltration of macrophages and lymphocytes. This result suggests that the autologous iPSC-derived neural cell transplants were not rejected by the primate brain [122]. Another study showed that transplantation of autologous iPSC-derived dopamine neurons into a non-human primate Parkinson’s disease model for up to 2 years provided functional recovery and immune tolerance without immunosuppression [123]. A similar result was published in the first iPSC-based clinical trial in RIKEN in 2017 where the authors transplanted an autologous iPSC-derived retinal epithelial cell sheet into a patient with neovascular age-related macular degeneration. The result indicated that the graft could survive more than two years after transplantation without immune suppression [11]. Together, these studies indicate that iPSC-derived cells may provide a new source for cell therapy.

#### Timelines and costs

Although there are obvious advantages to using autologous iPSC-based cell therapies, the pipeline of iPSC generation, characterization, and cell banking is a labor-intensive, highly time- and cost-consuming process. In general, it costs US $10,000–$25,000 to generate and validate a research grade iPSC line. The entire process requires between 6 to 9 months from patient recruitment to final characterization and requires further 3 to 6 months to produce large scale iPSC derivatives. Generating a clinical grade iPSC line costs approximately US $800,000 based on previously published reports [124, 125]. Therefore, to maximize the utility and efficiency of iPSCs and to significantly reduce the cost of generating an iPSC line, an alternative and practical strategy for personalized iPSC generation is to establish an allogenic iPSC resource for human leukocyte antigen (HLA)-matched tissue transplantation. Several similar projects have been started around the world since it has been proposed that 50 HLA homozygous “super donors” could match over 90% of the Japanese population [126]. A similar strategy, reported by Taylor et al., found that generating 150 selected HLA homozygous donors’ iPSCs could match 93% of the UK population [127].

#### Standardization

Variability within various iPSC lines and their derivatives remains a great concern when using iPSCs and their derivatives for disease modeling and cell therapy. Variability is often observed in iPSC differentiation potential, tumorigenicity, genome instability, epigenetic status, and maturation status within inter- and intra- iPSC lines when generated from different individuals and iPSC core facilities. The successful generation of “comparable” iPSCs and their derivatives relies on quality attributes to produce consistent, high-quality iPSCs. Thus, a QC guideline for producing clinical-grade iPSCs has been reported in 2018 by the Global Alliance for iPSC Therapies in the UK [128]. The critical quality attributes for clinical-grade iPSC generation include identity confirmation, microbiological sterility, endotoxin, genetic fidelity and stability (karyotyping and residual vector testing), potency determination, pluripotency marker expression, and post-thawed viability [128]. However, variations may still exist during iPSC expansion, reprogramming, colony selection, culture system selection, iPSC differentiation, and the purification process within different iPSC cell banks. Routine and continual validation of the iPSCs is required to solve such problems.

#### Genetic variations and stability

Recent studies of genetic and epigenetic variations in iPSCs raised concerns about safety in iPSC use. The presence of genetic variations in iPSCs includes genome instability, single nucleotide variations, CNV, and loss of heterozygosity. These mutations can be introduced and accumulated in iPSCs from their parental cells, reprogramming process, and generated during prolonged culture in vitro [129]. One safety concern about genetic variations in iPSCs is the possibility of tumorigenicity. The first clinical iPSC trial which treated age-related macular degeneration with an autologous iPSC-derived retinal pigment epithelial cell (RPE) sheet was conducted in 2014 in Japan [130]; however, Mandai et al. reported that three CNV were found in the second patient’s iPSCs and iPSC-derived RPE. Thus, the authors decided not to transplant the RPE sheet even if the iPSC-derived RPE passed the tumorigenicity test [11] despite there being no human iPSC-derivative clinical trials reporting the formation of neoplasia tissue after cell transplantation [11, 131, 132]. Moreover, it is known that various iPSC lines have different differentiation efficiency [133, 134]. Another concern for genetic and epigenetic variations among iPSCs is that variations may affect the iPSC differentiation potential and cause an unexpected phenotype of iPSC-derived cells [135–137]. The genetic variations in iPSCs may cause functional and safety consequences, thus, further studies and generation of a common iPSC-related mutation database and an established standard for screening of genetic variation are required for genomic stability evaluation.

#### Interspecies chimerism

Currently, researchers are attempting to use human iPSCs to generate interspecies chimeras. They aim to improve in vivo research models by generating human organs and tissues in animals or by generating new human disease models. Wu et al. (2017) reported that human iPSCs are capable of integrating into pig embryo [138]. However, there are still concerns in this field, for example, 1) for safety concern, the organ may be rejected by recipients even when receiving immunosuppressants during the xenotransplantation process; 2) serious zoonotic risks and contamination from animal cells when creating human-animal chimeras [139]; 3) the ethical issues, human-chimeric animals may have consciousness; 4) animal welfare issue, human cells may lead to unexpected suffer on chimeric animal [140]. Even though there are some advantages for this potential technique, the ethical issues for generating human-animal chimeras still requires further public discussion.

## Conclusions

The discovery of iPSCs has not only expanded our knowledge of the cellular mechanisms involved in pluripotency and development but has also allowed the opportunity for enhanced, human-specific drug screening and disease studies. These cells are becoming ever more prominent and continue to play a vital role in bringing more relevant cell models into the lab. Further advancement in iPSC technology will highlight their role in regenerative medicine. However, the cost and time required for the generation iPSCs remain ongoing roadblocks for many researchers. The continued development of iPSC banks provides a greater opportunity for researchers to gain access to these valuable cells while at the same time beginning to standardize their quality and reliability.

## Data Availability

The information for normal/disease iPSC lines are available in various iPSC repositories. Hyperlink for these repositories are listed below. CIRM: https://www.cirm.ca.gov CiRA: https://www.cira.kyoto-u.ac.jp/e/ FCDI: https://fujifilmcdi.com/the-cirm-ipsc-bank/ EBiSC: http://www.ebisc.org/ HipSci: http://www.hipsci.org/ Taiwan Human Disease iPSC Consortium: http://ipsc.ibms.sinica.edu.tw/index.html; https://catalog.bcrc.firdi.org.tw/Welcome RIKEN: https://cell.brc.riken.jp/en/hps/patient_specific_ips KSCB: http://www.cdc.go.kr WiCell: https://www.wicell.org

## Abbreviations

BCRC: Bioresource Collection and Research Center
BRC: BioResource Research Center
CiRA: Center for iPS Cell Research and Application
CIRM: California Institute for Regenerative Medicine
CNV: copy number variation
EBiSC: European Bank for induced pluripotent Stem Cells
ESC: embryonic stem cells
FIRDI: Food Industry Research and Development Institute
IBMS: Institute of Biomedical Sciences
IBMT: Fraunhofer-Institut für Biomedizinische Technik
iCeMS: Center for iPS Cell Research and Application
IMS: Information Management System
iPSC: induced pluripotent stem cell
KSCB: Korean Society for Cell Biology
QA: quality assurance
QC: quality control
RPE: retinal pigment epithelial cell
SNP: single nucleotide polymorphism
